# Coal

**Published:** 1874-08

**Authors:** 


### COAL.

When it is taken into account that some steamships consume a hundred tons of coal in twenty-four hours, and that five tons will supply an ordinary grate all winter, some might conjecture that the supply of coal will be exhausted and leave the world to freeze to an icicle.

But although the steamship consumes as much coal in a day as would feed a family grate for twenty years, and steamships, and furnaces, and forges, and manufactories consume millions of tons every twenty-four hours, yet there is enough coal in the United States alone to supply the globe for a thousand years. Fifty years ago "stove coal," as it was then called, was considered as worthless as a piece of slate; yet, thirty years ago, one million tons were dug out of the earth in Pennsylvania alone, and during 1873 twenty million tons. It is upon coal we must rely for healthful warming of our dwellings. Why not build a big fire at Pottsville and send the heat through underground pipes, and save the trouble, and waste, and expense of bringing the coal itself? Coal gas can be sent for miles under ground—why not heated air, which is only a purer gas?

